# The evolution of humanitarian mapping within the OpenStreetMap community

**DOI:** 10.1038/s41598-021-82404-z

**Published:** 2021-02-04

**Authors:** Benjamin Herfort, Sven Lautenbach, João Porto de Albuquerque, Jennings Anderson, Alexander Zipf

**Affiliations:** 1 Heidelberg Institute for Geoinformation Technology, 69120 Heidelberg, Germany; 2 GIScience Chair, Institute of Geography, Heidelberg University, 69120 Heidelberg, Germany; 3 Institute for Global Sustainable Development, University of Warwick, Coventry, CV4 7AL UK; 4 Alan Turing Institute, London, NW1 2DB UK; 5 University of Colorado Boulder, Boulder, CO 80309 USA

**Keywords:** Sustainability, Natural hazards, Computer science, Information technology

## Abstract

In the past 10 years, the collaborative maps of OpenStreetMap (OSM) have been used to support humanitarian efforts around the world as well as to fill important data gaps for implementing major development frameworks such as the Sustainable Development Goals. This paper provides a comprehensive assessment of the evolution of humanitarian mapping within the OSM community, seeking to understand the spatial and temporal footprint of these large-scale mapping efforts. The spatio-temporal statistical analysis of OSM’s full history since 2008 showed that humanitarian mapping efforts added 60.5 million buildings and 4.5 million roads to the map. Overall, mapping in OSM was strongly biased towards regions with very high Human Development Index. However, humanitarian mapping efforts had a different footprint, predominantly focused on regions with medium and low human development. Despite these efforts, regions with low and medium human development only accounted for 28% of the buildings and 16% of the roads mapped in OSM although they were home to 46% of the global population. Our results highlight the formidable impact of humanitarian mapping efforts such as post-disaster mapping campaigns to improve the spatial coverage of existing open geographic data and maps, but they also reveal the need to address the remaining stark data inequalities, which vary significantly across countries. We conclude with three recommendations directed at the humanitarian mapping community: (1) Improve methods to monitor mapping activity and identify where mapping is needed. (2) Rethink the design of projects which include humanitarian data generation to avoid non-sustainable outcomes. (3) Remove structural barriers to empower local communities and develop capacity.

## Introduction

Drawing lessons from what has been called the “immeasurability” of the Millenium Development Goals (MDGs)^1^, much attention has been given towards indicators and frameworks to monitor the United Nation (UN) Sustainable Development Goals (SDGs)^2^. Early calls for a “Data Revolution for Sustainable Development” identified the need for new institutions, actors, ideas and partnerships to successfully monitor progress towards the SDGs^3^. The Sustainable Development Solutions Network have emphasized the role of the often-neglected geo-spatial and geo-referenced data to disaggregate information for almost every proposed indicator^4^. Accordingly, new sources and partnerships are critical to fill data gaps for existing and yet to be developed indicators and might provide a complementary way to tackle the estimated gap of 1 billion US$ in funding for national statistical offices^4^. Consequently, it has been identified that baseline geospatial data that should be provided by national agencies are often not accessible, not up-to-date or not available in standard format^5,6^. Additionally, it is evident that effective humanitarian action and decision making rely on accurate, up-to-date spatial information^5^. More recently, the UN’s Sustainable Development Goals Report 2020 surveys the devastating impacts of the COVID-19 pandemic for the SDGs and highlights the increased importance of “data innovations” to support sustainability goals by closing important gaps in data for monitoring and reporting on the SDG targets and indicators^7^.

**Figure 1:**
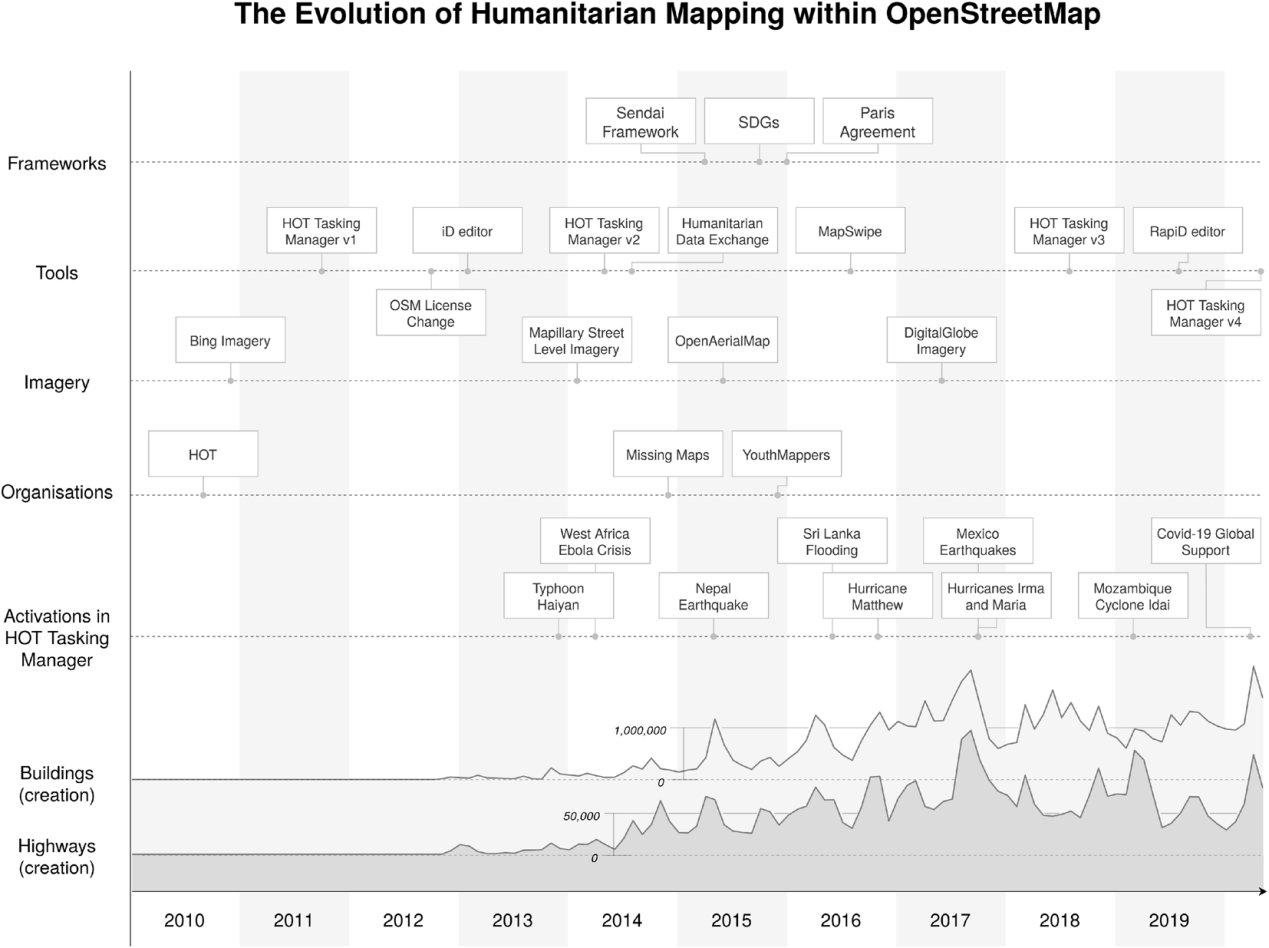
Sketch of the evolution of humanitarian mapping in OSM in regard to major disaster activations, the socio-technical development of the community and global political frameworks. The plots at the bottom show the number of buildings and highways added to OSM. Created using drawio 13.11.0 (https://github.com/jgraph/drawio).

It has been argued that several actions to achieve the SDGs (such as education, community engagement and community based problem solving, baseline research, planning and strategy development, allocation and coordination and improvement of public and private programs and services) can be informed by citizen generated data sets^8^. OpenStreetMap (OSM), also known as the Wikipedia of maps, constitutes a new open geographic database and a community mobilization initiative with potential to serve as a source of information for the called-for “data revolution”. The potential of OSM to harness the power of new information and communication technologies in support of disaster risk reduction has been recognized by several authors^9–11^. The past decade has seen the emergence of an ecosystem composed of volunteer mapping communities, corporations, governmental and humanitarian organizations which contribute to and use the open geographic database of OSM for various purposes. In this paper, we investigate the humanitarian mapping activity in OSM, predominantly organized through the web tool “HOT Tasking Manager”. While initially the term ’disaster mapping’ has been introduced to describe the “ability for volunteers to assist in disaster response situations via mapping and other spatial analysis”^12^, since 2010 the broader term ’humanitarian mapping’ has evolved. It refers to collaborative mapping in OSM (and other platforms such as MapSwipe or Ushahidi) for both humanitarian relief responses and humanitarian purposes in general^13–15^. This activity is also referred to as remote mapping or digitization and consists of the generation of geographic data based on satellite imagery^15^. Since contributors do not need to physically be at the place of mapping, the efforts are collaborative and distributed to volunteers around the world. The HOT Tasking Manager (https://tasks.hotosm.org/) has been used to coordinate the mapping efforts of thousands of volunteers by various organisations such as the Humanitarian OpenStreetMap Team (HOT), American Red Cross (ARC), and Médecins Sans Frontiéres (MSF), as well as collaborative projects such as Missing Maps (https://www.missingmaps.org/) or YouthMappers (https://www.youthmappers.org/) that particularly align their efforts to the SDGs. Figure 1 provides context and motivation for our research by showing a chronology of key events in humanitarian mapping since 2010, the year HOT was founded in the aftermath of the devastating Earthquake in Haiti^12,13^. It shows the immediate impact that post-disaster community “activations” have had on the contributions to OSM. Besides these punctual events, there are several additional factors with more long-term effects such as the introduction of new tools, the availability of open satellite imagery for mapping in OSM and the number of organizations involved. Furthermore, political frameworks set the policy agenda and form the institutional space in which local communities, national and international NGOs operate.

Previous work that analyzed humanitarian mapping in OSM has mostly focused on single events or relatively short time periods. For instance, there are detailed analyses of the mapping activity in OSM after the 2013 Typhoon Yolanda^13,14^, the 2014 West Africa Ebola response^14^, and the 2015 Nepal earthquake^16^. It has been shown that humanitarian remote mapping has undergone a series of changes in a socio-technological evolution between 2010 and 2013^13^. The 2015 Nepal earthquake has been identified as a turning point in the adoption of OSM by a wide range of organizations, leading to open-data practices becoming more common in humanitarian work and to the constitution of a community of practice^17^. The Missing Maps project created in 2014 has shifted the focus of large-scale humanitarian mapping from disaster response to preparedness and preventative efforts^10,14^. More recently, new actors have joined and existing mapping tools (such as the HOT Tasking Manager) are continuously improving with advanced technological approaches such as deep learning/artificial intelligence, which are now finding their way into humanitarian mapping workflows^18,19^. For a more detailed review of the scientific literature on this topic please refer to Haworth et al.^20^.

A key underlying motivation behind the rich and multifaceted efforts of OSM humanitarian mapping has been to widen the availability of its geographic database by creating the “missing maps” needed so “everyone is counted, map data is accessible and used, everyone can engage and contribute to the map.”^21^. This builds upon the acknowledgment that the OSM data in general is strongly biased, in part due to a much larger contributor basis in countries in the global North as a consequence of socio-economic inequalities and the digital divide^22^. However, the question arises to what extent humanitarian mapping efforts have been able to widen the footprint of OSM to overcome the data gaps caused by socio-economic inequalities 10 years after what could be considered the first large-scale humanitarian mapping event in the aftermath of the 2010 Haiti Earthquake. How much has humanitarian mapping contributed to making the world-wide collaborative map more inclusive to support the overarching SDG goal of “leaving no one behind”?

In order to tackle these questions, this paper presents a comprehensive assessment of the evolution of humanitarian mapping in OSM. This analysis encompasses all humanitarian mapping projects organized through the HOT Tasking Manager since 2012 (start of the available data), enabling us – for the first time – to cast a longitudinal perspective on the intersecting effects of mapping efforts, socio-economic, and demographic characteristics. This analysis provides critical insights into the achievements of humanitarian mapping so far, by combining data about OSM humanitarian mapping with statistics on global population distribution and human development at the sub-national level. Only very recent technological advances to utilize OSM’s full-history data for spatio-temporal data analysis on the global scale^23^ allow us to provide this novel perspective in our research. This spatio-temporal analysis is performed in the pursuit of the following research questions:

1. What is the effect of humanitarian mapping in the past decade on the global footprint of OSM in terms of socio-economic and demographic profile of the areas mapped?
2. How did OSM humanitarian mapping activity evolve over time on a global scale and in individual countries?

The remainder of this paper is organized as follows: The next section presents the results of our analysis of spatial footprint, temporal evolution and socio-economic characteristics of humanitarian mapping in OSM vis-‘a-vis general OSM mapping. This is followed by a discussion of our findings. The methodology is explained in the subsequent section and contains information on the data sets utilized and the statistical models applied.

## Results

### Scale and Spatial Footprint

Our results provide insight on the scale and spatial footprint of general mapping activity in OSM, which constitutes the baseline to evaluate humanitarian mapping efforts. As depicted in Table 1, all mappers (this term is often used to refer to the volunteers that edit the OSM database) have added 426.4 million buildings and 175.7 million highways in OSM between January 2008 and May 2020. Humanitarian mapping activities accounted for 60.5 million buildings (~14%) and 4.5 million highways (~3%). For buildings, changes to existing data in OSM, e.g. changing the shape of a building (geometry change) or changing its descriptive attributes (tag change) were observed less often in total and the proportion of humanitarian mapping varied between 4% - 12%. Highway mapping in OSM was characterized by frequent changes to existing data (geometry changes, tag changes).

**Table 1:** Cumulative number of added, changed and deleted buildings and highways in OSM between 2008/01/01 and 2020/05/20 in regard to overall and humanitarian mapping activities.

	Building contributions [millions]		Highway contributions [millions]	
	All	Humanitarian	All	Humanitarian
Added	426.4	60.5	175.7	4.5
Tag change	91.3	3.6	151.9	0.5
Geometry change	75.9	9.4	276.4	3.3
Deleted	32.6	3.2	23.2	0.3

**Figure 2:**
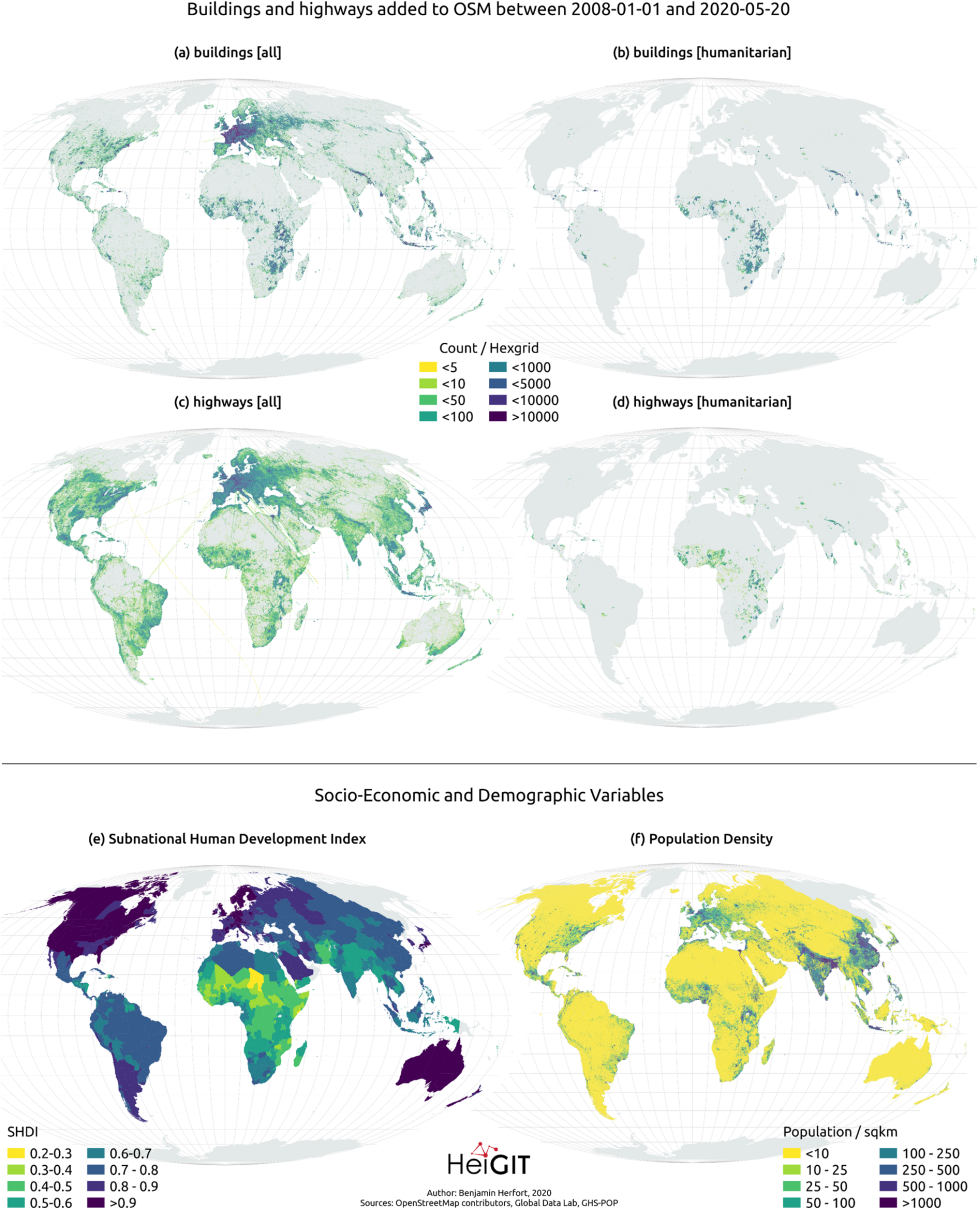
Spatial distribution of the number of (**a**)–(**b**) buildings and (**c**)–(**d**) highways added to OSM between 2008/01/01 and 2020/05/20 in regard to overall and humanitarian mapping activities. Further the maps depict the spatial distribution of (**e**) SHDI and (**f**) population density. Note that population count (and not density) is used in the statistical models. Created using QGIS 3.10.8 (https://www.qgis.org/en/site/).

Mapping of buildings in OSM predominantly happened in Europe, North America, and Japan (see Fig. 2). Furthermore, there has been considerable building mapping activity in Nepal, East Africa, the Philippines, and Indonesia (Fig. 2a). These were largely contributed through humanitarian mapping activities (Fig. 2b). Besides this, extensive humanitarian mapping activity were observed in Central and Western Africa, whereas activity was much more spatially scattered for Central and Southern America and Central Asia.

For highway mapping in OSM (see Fig. 2c), overall activity happened on a truly global scale in all inhabited parts of the world. Compared to the global reach of OSM mapping in general, humanitarian highway mapping activities (see Fig. 2d) focused on similar areas as the humanitarian building mapping activities and were extensive primarily in parts of Eastern and Western Africa. This map illustrates the observation that humanitarian highway mapping constitutes only a small fraction of overall highway mapping not only in terms of absolute numbers but also regarding its spatial distribution. Socio-economic data on the Sub-national Human Development Index (SHDI) and population distribution provide important variables to assess the scale and spatial footprint of overall and humanitarian mapping in OSM (see Fig. 2e,f). This is explored in the next section.

### Socio-economic and demographic characteristics

**Figure 3:**
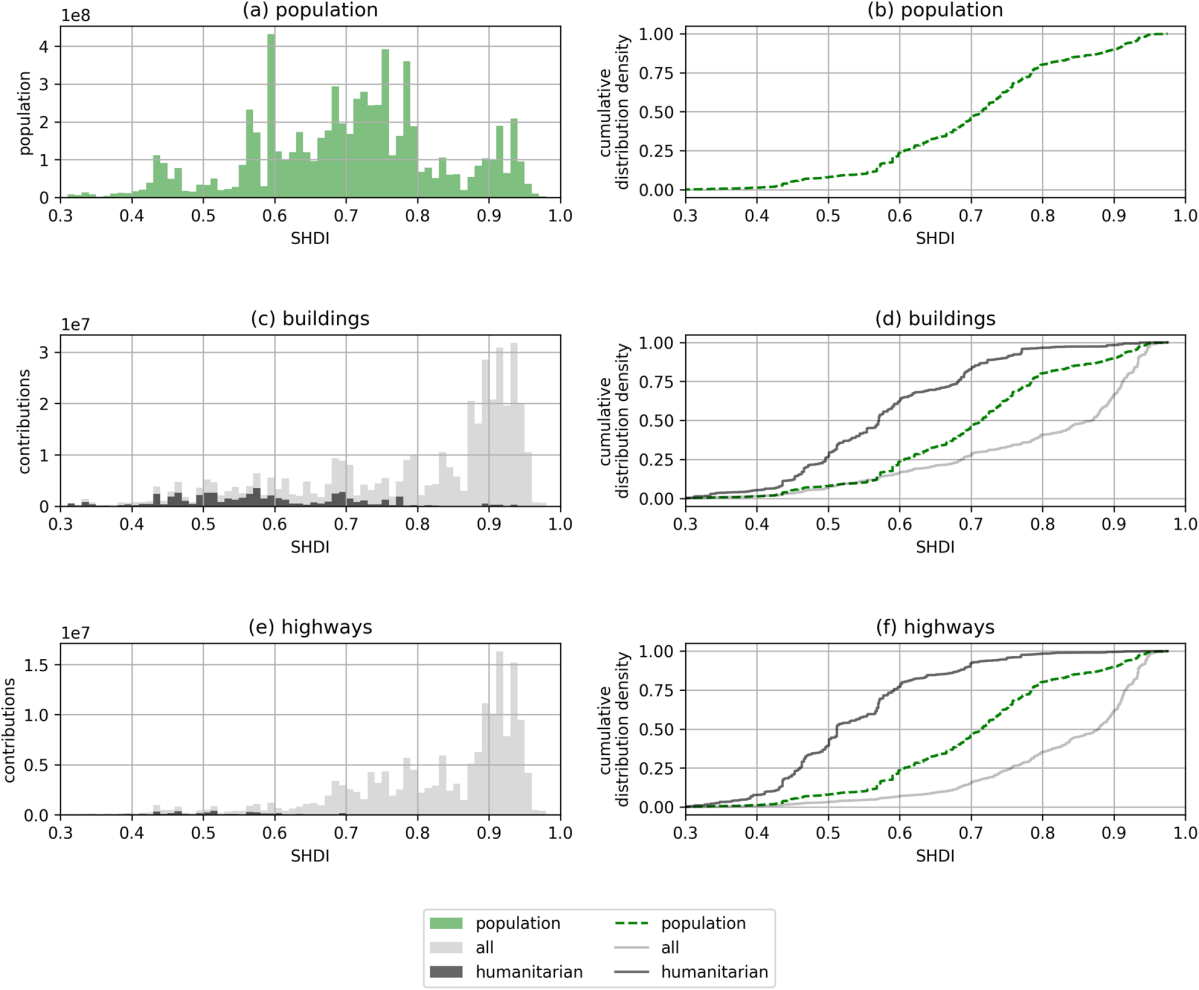
Frequency distribution and cumulative distribution density of (**a**)–(**b**) population and cumulative number of (**c**)–(**d**) buildings and (**e**)–(**f**) highways added to OSM between 2008/01/01 and 2020/05/20 in regard to to overall and humanitarian mapping activities and Subnational Human Development Index. Created using Matplotlib 3.3. in Python 3.7.5 (https://www.python.org/).

There was a sharp contrast between population distribution per SHDI (a) and mapping in OSM per SHDI for buildings (c) and highways (d) when considering the subnational level (Fig. 3). Whereas 20% of all humans lived in regions of very high human development, these regions accounted only for 60% of buildings and 65% of highways created in OSM. Regions with medium human development made up for 15% of added buildings and 11% of added highways in OSM in overall, but represented only ~36% of the global population. In regions with low human development we counted 10% of the global population, 11% of added buildings and 5% of added highways in OSM. Humanitarian mapping activities have been organized mainly in regions with an SHDI lower than 0.7. For humanitarian mapping, 83% of building creations and 92% of highway creations fell into this category.

The distinction between overall and humanitarian mapping activities has been underlined by the results of the Generalized Linear Model (GLM) regression both for buildings and in highways (Table 2). These results have been computed at the subnational level. In regard to buildings 12 respectively 8 spatial eigenvectors for overall and humanitarian contributions were included in the model, which underlines the complexity of the relationship. For highways the number of selected spatial eigenvectors were 16 for overall and 7 for humanitarian contributions. Spatial eigenvectors are additionally generated co-variates that account for the effect of spatial autocorrelation in the data^24^. They are based on the spatial weight matrix that defines neighborhood relationships between the observations. Without considering spatial autocorrelation coefficients were of same sign and order of magnitude (Table 2). The results revealed for both overall and humanitarian mapping an expected significant positive relationship between population (log scaled) and observed mapping in OSM for buildings and highways. Similarly, more buildings and highways have been added to OSM in regions that were impacted stronger by disasters (measured by the number of deaths caused by natural hazards per 100,000 inhabitants) for both overall and humanitarian mapping. The effect of the number of YouthMappers chapters was significant for the number of buildings but not for the number of highways for both overall and humanitarian mapping. Even for the number of buildings, the effect of the number of YouthMappers chapters was much weaker than for all other predictors.

The effect of SHDI on mapping activity differed clearly between overall OSM and humanitarian mapping activities: Considering overall mapping in OSM, we found a strong positive effect (more mapping in higher SHDI regions). For humanitarian mapping however, the results showed a negative effect (more mapping in lower SHDI regions). Similar, but less pronounced was the difference in regard to gender equality. Overall more buildings and highways have been mapped in regions with higher gender equality. Hence, gender inequality seemed to be a factor related to the prevention of mapping. But for humanitarian mapping this effect was less pronounced and could only be estimated with a higher uncertainty. This might indicate that humanitarian mapping mitigates the negative effects of gender inequality on OSM mapping at least to some degree. Cellphone usage showed an ambiguous effect on mapping activity. For highways mapping its effect was very low and not significant at a level of 0.01. However, it seems that there was a stronger positive effect on humanitarian building mapping. In our model, cellphone usage was the variable which showed the highest collinearity with SHDI. This might have contributed to the observed higher uncertainty of the regression coefficient estimates. In total, our models explained 79% (buildings) and 91% (highways) of the observed deviance for overall OSM mapping activity. With regard to humanitarian mapping activity, we observed an explained deviance of 68% and 72%. Explained deviance for models without spatial eigenvectors were 60% and 83% for all OSM and 54% and 60% for humanitarian mapping. The increase in explained deviance shows that the spatial eigenvectors presumably captured effects of non available covariates with spatial structure that would otherwise lead to induced spatial autocorrelation in the regression residuals.

Analyzing the residuals of the GLM emphasizes the regional variations in humanitarian mapping activity. The residuals tell for which regions the association between response and predictors differed from the general relationship across all subnational units. The residuals have been derived for the subnational units and aggregated in a subsequent step to allow an interpretation on the country level. Hence, the analysis highlights most deviating countries (and not subnational units) where either other factors not captured in our analysis drive or prohibit mapping, or the effect of our set of predictors on mapping was considerably different to the global coefficients. The spatial eigenvector mapping captured some of the spatial pattern present in the data. Hence, residuals provide insights on deviations that were beyond what could be explained by the spatial structures in the data itself.

**Figure 4:**
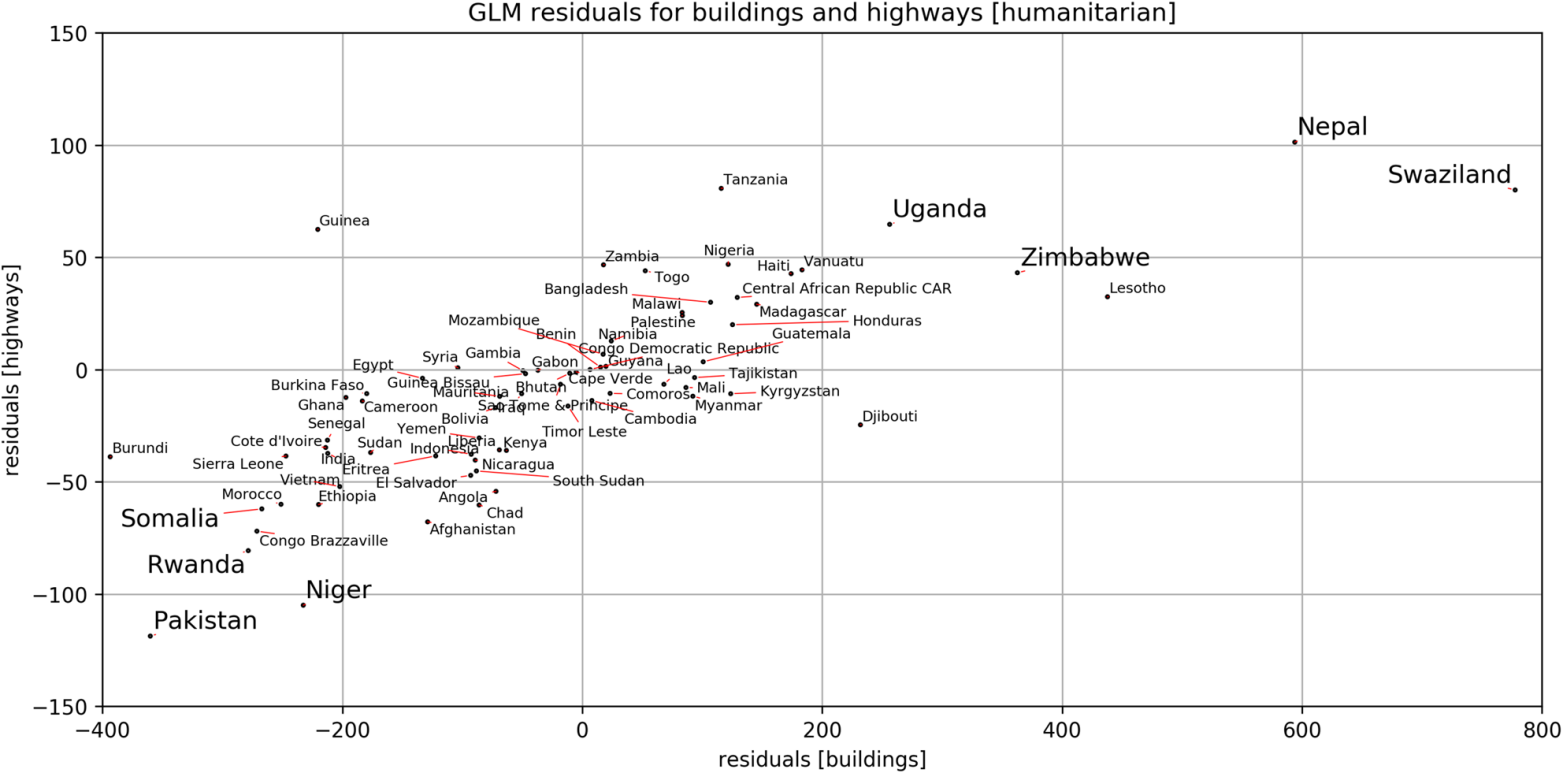
Distribution of GLM residuals for the number of buildings and highways added to OSM between 2008/01/01 and 2020/05/20 in regard to humanitarian mapping activities. The residuals have been derived for the subnational units and have been aggregated to the national level in a subsequent step. This plot depicts the values for all countries with medium and low human development (SHDI<0.7). Countries mentioned in the text are highlighted by increased font size. Created using Matplotlib 3.3. in Python 3.7.5 (https://www.python.org/).

Figure 4 provides an overview on the residuals of the GLM in regard to humanitarian buildings and highways contributions for all countries with low and medium human development (SHDI< 0.7). Countries for which both residuals were negative showed stark, less-than-average humanitarian mapping contributions for buildings and highways. Among these countries were Pakistan, Somalia, Rwanda and Niger. For these regions we suspect the existence of factors which prevented mapping.

Zimbabwe and Uganda were part of the countries for which we observed humanitarian contributions slightly above average. For Uganda the residuals indicated the existence of moderately boosting factors, such as funding for mapping activity in Kampala provided from the Open Cities program as part of World Banks Open Data Resilience Initiative^25^.

Finally, Nepal and Swaziland have been identified as outliers with unexpectedly high humanitarian contributions for buildings and highways. As depicted already this underlines the impact of the mapping in response to the 2015 Gorkha Earthquake in Nepal^16^. In Swaziland, extensive mapping has been organized by HOT during the Malaria Elimination Mapping Programme in 2016.

**Table 2:** Results of Quasi Poisson Generalized Linear Regression Models with and without Spatial Eigenvector Mapping for cumulative number of buildings or highways added to OSM in regard to to overall and humanitarian mapping activities.

	All			Humanitarian		
	Estimate	Std. err.	z value	Estimate	Std. err.	z value
**Buildings, SEVM**						
(Intercept)	− 12.33***	0.62	− 19.79	− 15.62***	1.22	− 12.77
log(population)	13.24***	0.33	40.37	13.75***	0.64	21.56
shdi	0.41***	0.04	10.83	− 1.13***	0.08	− 14.29
cellphone users	0.11**	0.03	3.26	0.26***	0.06	4.06
gender equality	0.21***	0.03	7.30	0.15**	0.06	2.62
disaster deaths	0.39***	0.04	9.01	0.28***	0.05	5.51
youthmappers	0.03**	0.01	2.77	0.04*	0.02	2.16
	explained deviance: 0.79			explained deviance: 0.68		
	Eigenvectors: 12			Eigenvectors: 8		
**Buildings, without SEVM**						
(Intercept)	− 10.93***	0.77	− 14.12	− 17.23***	1.20	− 14.40
log(population)	12.56***	0.40	31.02	14.79***	0.62	23.88
SHDI	0.62***	0.05	13.72	− 1.27***	0.07	− 17.56
cellphone users	− 0.003	0.42	− 0.09	0.27***	0.05	5.14
gender equality	0.38***	0.03	11.29	0.34***	0.05	7.19
disaster deaths	0.58***	0.05	12.17	0.19***	0.05	3.57
youthmappers	− 0.007	0.02	− 0.43	0.03	0.02	1.62
	explained deviance: 0.60			explained deviance: 0.54		
**Highways, SEVM**						
(Intercept)	− 11.78***	0.38	− 31.11	− 20.06***	1.43	− 13.99
log(population)	12.32***	0.20	61.44	14.54***	0.74	19.65
SHDI	1.03***	0.03	35.46	− 1.29***	0.09	− 14.60
cellphone users	0.04*	0.02	2.04	0.03	0.07	0.056
gender equality	0.12***	0.02	7.32	0.15*	0.06	2.52
disaster deaths	0.21***	0.05	4.32	0.37***	0.05	7.17
youthmappers	− 0.01	0.001	− 1.05	0.04	0.02	1.86
	explained deviance: 0.91			explained deviance: 0.72		
	Eigenvectors: 16			Eigenvectors: 7		
**Highways, without SEVM**						
(Intercept)	− 9.56***	0.43	− 22.35	− 23.11***	1.54	− 15.00
log(population)	11.10***	0.22	49.49	16.33***	0.79	20.65
SHDI	1.10***	0.03	36.21	− 1.56***	0.10	− 16.20
cellphone users	0.07**	0.03	2.74	0.11	0.07	1.64
gender equality	0.14***	0.02	7.21	0.46***	0.06	8.18
disaster deaths	0.32***	0.05	6.34	0.28***	0.06	4.76
youthmappers	− 0.02*	0.01	− 2.07	0.003	0.03	0.14
	explained deviance: 0.83			explained deviance: 0.60		

The spatial eigenvectors contribute to the explained deviance. For models without incorporation of spatial autocorrelation standard errors are too small, coefficients might be biased - these models are only reported for comparison. Regression coefficients are reported for standardized predictors to make the effect of the different predictors comparable. The only exception is population which has not been standardized due to the use of the log-transformation and since population is included as a covariate that needs to be controlled for but not of primary interest. Significance levels: $$^{*}P<= 0.05, ^{**}P<= 0.01, ^{***}P <= 0.001$$.

### Temporal evolution

**Figure 5:**
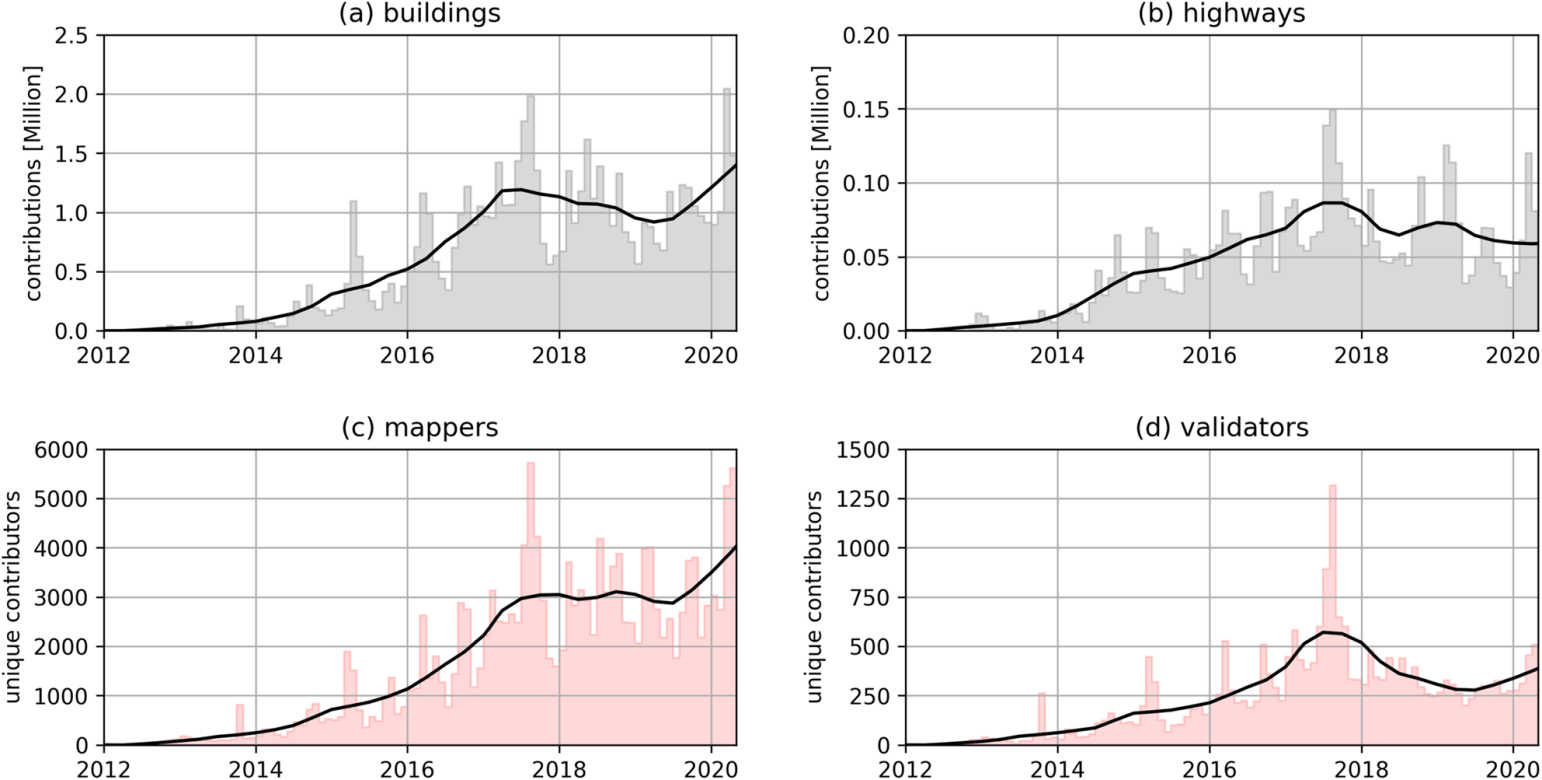
Monthly frequency distribution and trend cycle of humanitarian OSM contribution counts between 2012/01/01 and 2020/05/20 for (**a**) buildings and (**b**) highways and (**c**) mappers and (**d**) validators in HOT Tasking Manager. The line plots provide the trend line for each variable. Created using Matplotlib 3.3. in Python 3.7.5 (https://www.python.org/).

This section draws attention to the temporal evolution of humanitarian mapping activity in OSM. Figure 5 provides monthly activity and trend-cycle for the number of (a) buildings and (b) highways added to OSM and further shows the number of volunteers in the HOT Tasking Manager that added data to OSM (mappers) and the number of volunteers that reviewed and corrected the work of others (validators).

The peaks in the time series plots revealed the impact of major disaster activations and organized mapping campaigns. During the mapping activations for 2013 Typhoon Haiyan and 2014 West Africa Ebola Crisis, up to 1000 mappers added nearly 250,000 buildings and 40,000 highways to OSM per month. The mapping efforts after the 2015 Nepal Earthquake were supported by up to 1900 mappers in the immediate aftermath of the disaster. In the month of the activation more than 1 million buildings and around 70,000 highways were added to OSM through the HOT Tasking Manager. The mapping activations for the 2017 Mexico Earthquake—as well as Hurricanes Irma and Maria around the same time—have resulted in unprecedented high numbers. More than 5700 mappers added around 2 million buildings and 150,000 highways. Subsequent disaster activations, such as the response to 2019 Cyclone Idai in Mozambique showed fewer engagement. Only recently, the mapping activities during the COVID-19 pandemic reached the highest level from 2012–2017. In April 2020, more than 5000 users added more than 2 million buildings and 120,000 highways.

Besides the monthly peaks related to disaster response efforts, humanitarian contributions in OSM have also increased in times without major disaster activations. The trend-cycle analysis revealed that humanitarian mapping has experienced sustained growth regarding monthly added buildings and highways from 2012 to mid-2017. This was supported by a growing number of monthly active mappers and validators participating in the HOT Tasking Manager. However, the trend-cycle analysis further depicted a consolidation phase since mid-2017. From this time on humanitarian building and highway mapping have shown stagnation or a slight downward trend. These trends were again supported by activity in the HOT Tasking Manager. Since mid-2017 there has been a stagnation in regard to number of mappers and a considerable decrease in number of validators. However, this downward trend might have changed again as the number of added buildings, mappers and validators increased since mid-2019.

**Figure 6:**
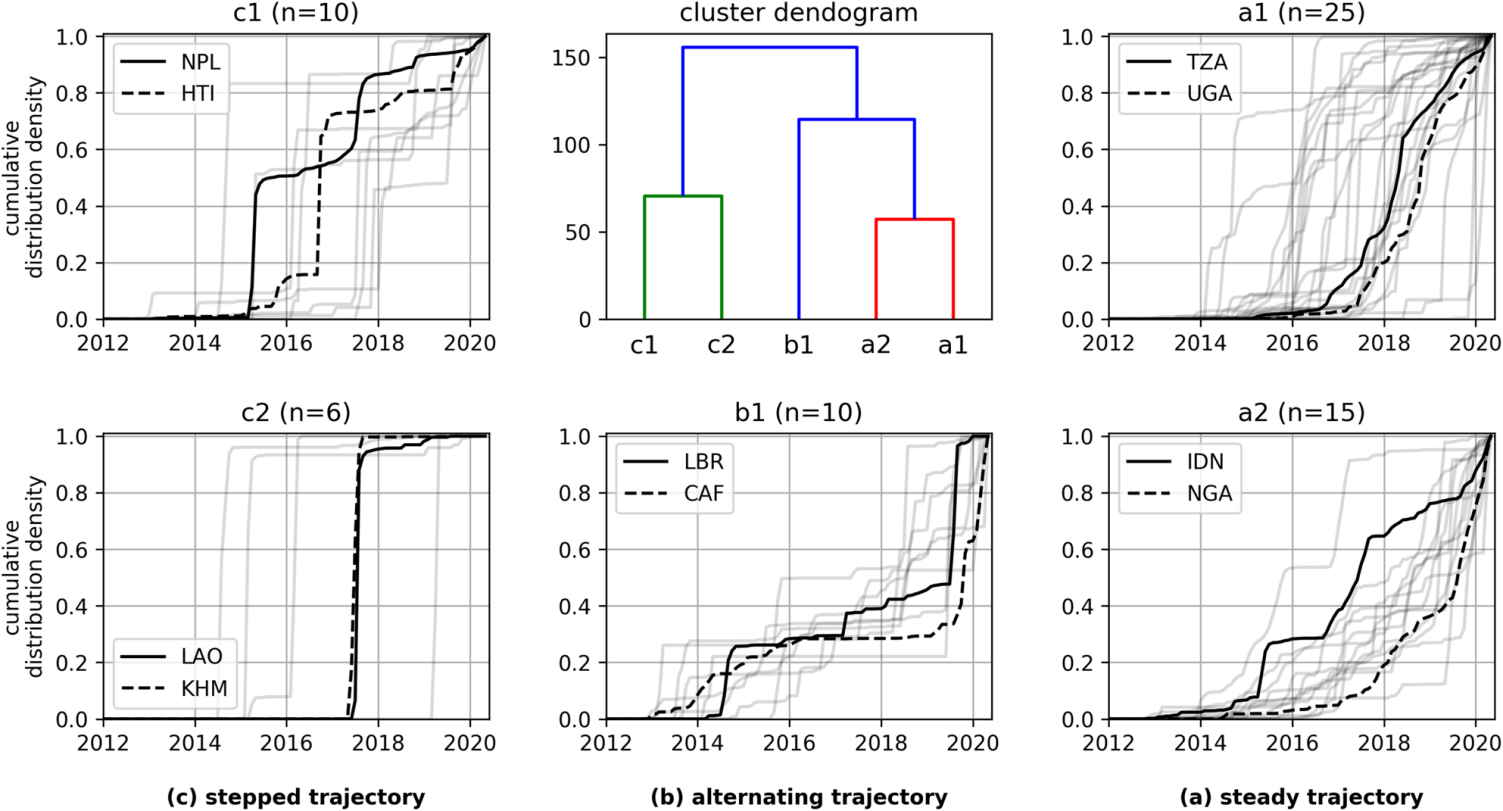
Cluster dendogram and cumulative distribution density plots for monthly humanitarian OSM building mapping between 2012/01/01 and 2020/05/20. This plot depicts the values for all countries with medium and low human development index (SHDI<0.7). The groups are defined by a hierarchical clustering approach based on a distance matrix derived from dynamic time warping time series similarity analysis. For each group the trajectory for the two countries with highest overall mapping activity are highlighted. We used alpha-3 ISO 3166 codes to refer to the countries: TZA - Tanzania, UGA - Uganda, NPL - Nepal, HTI - Haiti, LAO - Laos, KHM - Cambodia, LBR - Liberia, CAF - Central African Republic, IDN - Indonesia, NGA - Nigeria. Created using Matplotlib 3.3. in Python 3.7.5 (https://www.python.org/).

The previous results show that there were considerable variations between the extent and quantity of overall and humanitarian mapping in OSM, as well as changes in regard to the global temporal evolution of humanitarian mapping. This section explores the similarity between the number of monthly added, changed and deleted buildings in OSM per country utilizing a dynamic time warping approach. The analysis has been performed for all countries with medium and low human development. Based on agglomerative hierarchical clustering^26^ of the temporal evolution per country, we defined five groups which were part of three clusters: (a) steady trajectory, (b) alternating trajectory, (c) stepped trajectory. Figure 6 displays the cluster dendogram and the cumulative distribution density for each group. Table 3 provides further insights on the cluster association of all countries with low and medium human development.

Most countries showed a steady trajectory of humanitarian building mapping in OSM over time (cluster a). The biggest group of countries within this cluster (a1) contained 25 countries for which mapping has followed a three stage process. The trajectories for Tanzania and Uganda were exemplary for this group. Firstly, building mapping has grown slowly in these regions over a longer time. In the second stage mapping activity increased heavily, but steadily over a time of several months. Finally, in the last stage mapping activity declined, but stayed at a higher level compared to the time before the peak. In the cumulative distribution density plot this results in a S-shaped graph reminding of a logistic curve.

The trajectories for 15 countries being part of group (a2) also showed continued and steadily increasing mapping activity over time. However, the cumulative distribution density plots for most of these countries did not level off. Currently, many of these countries appear to be approaching a peak in mapping activity, which some may have already reached by the end of 2019.

Not all countries show an unambiguous temporal pattern. For most of the 10 countries in group (b1) humanitarian building mapping has started relatively early between 2013 and 2015. However the mapping trajectory alternated between times of steady mapping, times of accelerated mapping and also times when little mapping happened. Mapping in Liberia and the Central African Republic could be described in this way. As depicted in the cluster dendogram (see Fig. 6) this group could be seen as a hybrid between (a) and (c).

The third cluster (c) depicts 16 countries for which the cumulative distribution density plot revealed a trajectory with either one single or multiple characteristic steps per country. For the 10 countries in group (c1), this represented multiple sequences of times with relatively high mapping activity (e.g. during a disaster activation) followed by times with lower mapping activity (e.g. after an activation). The humanitarian mapping contributions in Nepal and Haiti followed this temporal pattern. Note that the Haiti mapping activity here is in responses to the 2016 Hurricane Matthew mapping activation and does not include the mapping activity from 2010.

Countries that have been subject to large-scale, one-time mapping efforts form group (c2). In the cumulative distribution density plot, this is characterized by shapes with one single, big step. For these countries almost all data has been added to OSM within a single short-term period. Only very little mapping happened before and after that time span. Laos and Cambodia are part of this group.

**Table 3:** Clusters of temporal trajectories of the monthly number of buildings added, changed and deleted in OSM per country between 2012/01/01 and 2020/05/20.

Cluster	Temporal evolution	Countries
(a)	(a1) steady - declining	Tanzania, Uganda, Zimbabwe, Mozambique, Bangladesh, Myanmar, India, Malawi, Mali, Guinea, Sierra Leone, Lesotho, Swaziland, Guatemala, Togo, Vietnam, Benin, Yemen, Djibouti, Niger, Ghana, Somalia, Tajikistan, Nicaragua, El Salvador
	(a2) steady - constant	Indonesia, Nigeria, Congo Democratic Republic, Zambia, Kenya, South Sudan, Burkina Faso, Madagascar, Chad, Honduras, Burundi, Cote d’Ivoire, Iraq, Kyrgyzstan, Ethiopia
(b)	(b1) alternating	Liberia, Central African Republic CAR, Cameroon, Senegal, Namibia, Gambia, Afghanistan, Sudan, Pakistan, Mauritania
(c)	(c1) stepped - multiple	Nepal, Haiti, Rwanda, Angola, Bolivia, Syria, Timor Leste, Congo Brazzaville, Bhutan, Egypt
	(c2) stepped - single	Laos, Cambodia, Palestine, Vanuatu, Comoros, Guyana

The groups are defined by a hierarchical clustering approach based on a distance matrix derived from dynamic time warping time series similarity analysis. This table lists all countries with medium and low human development index (SHDI<0.7).

## Discussion

The long-term, global perspective of our analysis has – for the first time – characterized and quantified humanitarian mapping’s significant impact on the volume and spatial footprint of objects added to OSM. We showed that there still exists a clear mismatch between the distribution of mapping activity in OSM vis-à-vis the distribution of the global population on the Earth’s surface. The unprecedented detail of our analysis allowed us to identify distinct areas of concentrated mapping efforts and areas for which more mapping is still needed such as South America, Central America and Central Asia. The GLM models showed that socio-economic and demographic characteristics were able to explain a large part of this mismatch and that humanitarian mapping has helped change the spatial footprint of OSM globally in regards to the socio-economic profile of areas mapped.

Furthermore, the analysis of the GLM residuals provided insights into the variability in humanitarian OSM contributions per country that could not be explained by socio-economic and demographic characteristics alone. This analysis makes it clear that there can be underlying factors that may promote (e.g. the occurrence of disasters) or block mapping (e.g. gender inequality), but which have not completely been captured in our analysis. These results have been refined by the analysis of the temporal evolution which enabled us to identify three groups with distinct profiles of OSM humanitarian mapping trajectories. This differentiation between (a) steady trajectory, (b) alternating trajectory and (c) stepped trajectory provides novel insights towards a typology of the temporal evolution of OSM contributions at the country level, complementing previous research analyzing trajectories of OSM communities^27,28^.

In line with the latest research presented at the OSM community’s 2020 State of the Map Conference^29^, our results confirm that mapping activity is strongly affected by disasters and seems to be positively influenced by the existence of an established local OSM community, local offices of the HOT, as well as the focus of third party projects such as Open Cities Africa funded by the World Bank^25^. However, the existence of YouthMappers chapters^30^ didn’t show a significant effect in our models. Further research should investigate to what extend YouthMappers chapters might focus more on local mapping in contrast to remote mapping of buildings and highways. In contrast, we suspect that in some countries (e.g. Pakistan^31^), local restrictions that are not captured in our model such as legal constraints to surveying and mapping might have a strong negative impact on the mapping activity.

The increasing contribution of corporate mappers^32^ is another pressing field where a long-term monitoring and fine grained understanding of temporal and spatial components of mapping in OSM is needed. In this regard, recent technical developments towards machine learning based methods to extract buildings and roads from very high resolution satellite imagery that have been promoted by companies (such as Facebook and Microsoft) and scientists are showing their impact on OSM already today^19,33^.

When it comes to the temporal evolution of OSM humanitarian mapping activity, our results could confirm the findings of other authors on the potential of collaborative mapping for disaster relief and the evolution it has gone through^13,14,16^. But this analysis also shows that the growth of the humanitarian mapping community faced challenges as indicated by a phase of reduced activity which started in 2017 and seemed to have ended only recently in the year 2019. To the best of our knowledge, this has not been addressed by other authors so far. The stepped-trajectory temporal patterns on the country level put into question the long-term effects of some organized mapping campaigns towards making OSM more inclusive and democratic. Some researchers have put this absence of mapping activity into context with the diminishing feeling of urgency and crisis which have been observed in the aftermath of this major disaster activations^34^.

The last months analyzed in this research fell into the time of the global COVID-19 spread. The overall temporal trend might indicate that this has contributed to increased mapping. However, investigating the further impact of COVID-19 on global OSM contributions had to remain out of scope for this research.

Unavoidably, our analysis comes with several limitations that need to be considered to put our findings into context. Our insights about humanitarian mapping in OSM only provide an incomplete picture which lacks an on-the-ground perspective and neglects other remote mapping tools, since we considered only the mapping that was organized through the HOT Tasking Manager. For instance, humanitarian mapping that has been organized by local residents on the ground is not considered here. This limitation is accompanied by the fact that our analysis only focused on two types of mapped objects (buildings, highways). Mapping in OSM comes with a much greater variety of potential map objects (e.g. health facilities, schools, water points), which can add particular value in comparison to other geographic data sets. We are aware of the fact that our definition of humanitarian mapping is therefore oversimplified and the results must be taken with a grain of salt. In many regions of the world there is no clear distinctive line between humanitarian and non-humanitarian mapping activities as the humanitarian and non-humanitarian OSM communities are not disjoint. Hence, not every contributor that used the HOT Tasking Manager is exclusively a “humanitarian” mapper, but might identify herself primarily as an OSM mapper or member of a local OSM community. Future work could expand this analysis with in-depth analyses of specific communities (e.g. using mixed qualitative-quantitative methods), as well as could investigate the role of other organized mappers such as cooperate mappers^32^ to disentangle the impact of the different sub-communities of OSM on mapping activities.

The observed explained deviance value in regard to overall highway mapping of 0.9 is very high and should be critically assessed as another potential limitation of our study. For both the distribution of buildings and roads a strong positive association with population density as well as with human development can be assumed. If one of the features would be mapped more completely, this is likely to lead to a stronger association compared to a feature that is less completely mapped. Roads have been the first feature mapped in many parts of the world and the road network has been estimated as  80%^28^ complete at the global scale. A comparative completeness analysis for buildings is missing. However, the differences in model performance might hint towards a higher completeness for roads. In addition, one has to keep in mind the presence of strong spatial patterns that are reflected by the spatial eigenvectors which increase model performance. In addition to the removal of spatial autocorrelation and of the associated problems in regression coefficient estimation^35^ this presumably also captured the effect of missing spatial co-variates. The increase in explained deviance for highways by incorporating the spatial eigenvectors was only modest for all OSM (from 83 to 91%). For the other three models the increase in explained deviance was stronger (12–19% absolute increase in explained deviance). This can be interpreted as that mapping of the OSM road network in countries with a high HDI was less influenced by prohibitive or stimulating factors.

Further limitations concern the information used from GHS-POP and SHDI which are only able to provide imperfect proxies for the actual population distribution and socio-economic profiles of regions. There are a few other data sets, such as WorldPop, that provide information on human population on the global scale^36^. Nevertheless, even such very high resolution datasets have similar limitations, i.e. the estimation of inhabited areas is biased towards urban areas^37^. As a consequence, settlements in rural areas are more likely to be left out in the global population data sets as well. The SHDI provides only a narrowed view on socio-economic development. Investigating the impact of the individual components of the SHDI (health, education, income) should be considered for future work. Information on cellphone users, gender equality, disaster deaths and YouthMapper chaperts were only available at the national scale. It has to be assumed that these vary as well between the subnational units. This has to be expected especially for disaster deaths. Adding further indicators to the analysis, such as the Multi Dimensional Poverty Index, could provide a way to better account for the structural effects that pose a challenge especially on poor and vulnerable populations^38^. In future work, we would like to investigate how this might reduce the uncertainty of the statistical models applied in this paper.

In conclusion, humanitarian mapping efforts of the previous decade have already made OSM more inclusive, contributing to diversify and expand the spatial footprint of the areas mapped. However, humanitarian organisations, local communities and other stakeholders involved should learn from the various challenges we have highlighted in this paper, in order to have sustained impact in the future. Based on our research results we want to highlight three important recommendations:

First: Improve methods to monitor mapping activity and identify where mapping is needed. At the current state for most organizations “monitoring” merely consists of counting the overall number of buildings and highways and usually neglects the spatial and temporal components of mapping activity. The humanitarian OSM community should therefore adopt monitoring methods that can more clearly address the question of “Where is mapping needed?”, instead of “How much mapping happened?”. The spatial analysis methods presented in this paper can offer a basis for starting addressing the former question.

Second: Rethink project design to avoid non-sustainable outcomes. We would recommend that the organizations that have been engaged in countries with one-time mapping efforts, e.g. in Laos or Cambodia, should be encouraged to assess how projects could be designed to contribute to more sustainable outcomes. We argue that “putting countries on the map” should be seen as only the first step towards considering such countries effectively included, as real inclusion requires an active local OSM community with the capacity to generate, maintain and improve geographic data that reflects local perspectives in the long term. Especially in the fast developing domain of applying artificial intelligence and machine learning methods to humanitarian mapping purposes, we see a critical lack of local engagement by design. This might reproduce the very same one-time-mapping patterns that have been observed in the past as revealed by our analysis.

Third: Remove structural barriers to empower local communities and develop capacity. Future mapping efforts should promote local data generation methods that not only achieve high-quality data but also simultaneously empower local communities and support them to acquire new perspectives on their territories and development potentials^39^. Focusing on empowerment goes beyond technically involving local communities in the map making process. In order to achieve this, humanitarian organisations and local OSM communities need to indentify the structural barriers that exclude some social groups from participating in (humanitarian) OSM mapping and work with them to develop capacity to overcome those barriers.

Balancing the unequal spatial and temporal contribution patterns in OSM clearly goes beyond the population of a more comprehensive geographic data base. It is about building diverse and vibrant communities that can take part in the map making process and are empowered by the mapping results. We believe that such an inclusive OSM community can fuel the much needed data revolution and will prove its value to inform better political actions towards achieving the SGDs.

## Methods

### Data

#### OpenStreetMap history data set

Our analysis evaluated data contributed to OSM by utilizing the OSHDB^23^ a high-performance spatio-temporal data analysis platform for OSM full-history data. Following this approach enabled us to investigate all edits to the OSM database and we refer to these as contributions. The OSHDB distinguishes between different aspects of user contributions: Whenever a users adds a new object in OSM (e.g. draws a polygon, sets the key-value pair to building=yes and uploads to OSM) this is referred to as a creation in OSM. Users can modify existing objects in OSM. If the attribute description of an object is adjusted (e.g. a users changes the value of highway=unclassified into highway=residential) or new attributes are added or existing ones are removed this is referred to as a tag change. Users can also modify the shape of existing objects in OSM. This so-called geometry change can be done by moving nodes (for all feature types node, way and relation) or by adding or removing nodes (for feature types way and relation). A change in the geometry will most likely cause a change (increase or decrease) in derived statistics such as length or area. Whenever an object is removed from the OSM database or the respective tag is removed from the object (e.g. “building” key is removed for a building, but the nodes are not deleted) this is referred to as a deleted object (deletion).

Our analysis covered contributions related to the following tag-value combinations and respective feature types in OSM: building=* (ways) and highway=* (ways). These feature types include all types of roads and paths as well as all types of buildings. For all objects we derived the number of contributions (count) and the payload (area in square meter for buildings, length in meter for highways). To allow for an unambiguous comparison between different contribution types (e.g. creation vs. tag change) and between different features (e.g. buildings vs. highways) we decided to report only on counts in the scope of this paper - instead of the common way of reporting the number of buildings and the length of highways.

The spatial unit of our analysis has been defined by a grid network of hexagons which has been generated using the DGGRID software^40^. All hexagons had the same size in terms of area and cover ~96 square kilometers (this refers to the so-called zoom level 12). Their spatial characteristics make hex grids an unit suited better for spatial analysis compared to other grid systems such as the Tile Map Service grid (which is handy to be displayed at web maps, but not so for deriving area based statistics).

#### HOT tasking manager data set

Our definition of humanitarian mapping activities utilized data from the HOT Tasking Manager platform which is online at tasks.hotosm.org. We were provided with a dump of the HOT Tasking Manager database by HOT covering all projects created within 2012–11 and 2020–05.

We refer to projects as defined by the HOT Tasking Manager. A project is usually set up by an organisation (e.g. American Red Cross) and defined by a number of tasks, which will be generated from a polygon geometry depicting the area of interest. Per project, project managers add information on which features (e.g. buildings, highways, waterways) should be mapped and which satellite imagery should be used for their digitization. Often the description will also provide contextual information for what purpose map data is collected.

Tasks are a central concept of the HOT Tasking Manager. For each project the overall area is split into many smaller areas (usually squares) which are defined as tasks. It’s common that several users will work on a task (e.g. user A maps it and user B validates it), but two users will never work on the same tasks at the same time in the HOT Tasking Manager. Hence, after a user selected a task it is locked (for maximum 2 h).

We measured the activity in the HOT Tasking Manager based on the concept of sessions, which we introduced for this research. A session has been defined by a user performing an action on a task. The action performed can be one out of the following which indicate the kind of change that the user induced: unfinished, mapped, validated, invalidated, split or bad imagery. For each session there was a start timestamp (the time the users selects the task to map in the HOT Tasking Manager) and an end timestamp (the time the user finished mapping and sets the status in the HOT Tasking Manager to either mapped or one of the others mentioned above). For each session we queried the OSHDB to calculate the OSM based statistics for buildings and highways contributed by the user for the given extent and time range.

#### Socio-economic and demographic datasets

Global population distribution played a central role in our research. We used the GHS-POP database^41^ which relies on the Global Human Settlement Layer (GHSL) provided by JRC and a disaggregation of CIESIN’s Gridded Population of the World (GPWv4.10). For the results presented here we used population information provided at a spatial resolution of 250 m for the year of 2015. We selected this data set instead of the data available from WorldPop due to the fact that the methodology applied by the team behind World Pop uses various geospatial covariate layers derived from OSM data. This could have introduced an unintended bias when comparing that data set with OSM. Since the statistical analysis performed in this research was targeted at the sub-national level GHS-POP based on GPW provides a solid basis.

To characterize regions based on their socio-economic status we utilized the Subnational Human Development Database^42^. This data set breaks down the national Human Development Index (HDI) based on data from statistical offices and aggregated census data and data derived from household surveys into their subnational counterparts. In our analysis we relied on the aggregated SHDI and did not further consider its individual components (education, standard of living, health).

We used information on the total number of deaths caused by disasters caused by natural hazards per 100,000 inhabitants provided by EM-DAT (International Disaster Database)^43^. We considered events which occurred for the years 2012 to 2020. The data is provided on the national level.

Gender equality has been selected as another co-variate in our analysis. Here we use the proportion of seats held by women in national parliaments (referring to SDG indicator 5.5.1). The data has been extracted from the United Nations’ Global SDG Indicators Database (https://unstats.un.org/sdgs/indicators/database/).

Information on cellphone usage (mobile cellular subscriptions per 100 people) has been derived from World Bank’s DataBank (https://databank.worldbank.org). The latest available information was used per country.

Finally, the number of YouthMappers chapters per subnational unit has been derived from the public chapter listing on their website (https://www.youthmappers.org/chapter-listing).

### Statistical analysis

#### Scale and spatial footprint

The spatial footprint of overall and humanitarian mapping activities is generated on the basis of the hexagonal grid described above. For each hexagon we derived the count of OSM contributions by intersecting the geometry of the OSM entity with the hexagon geometry. Humanitarian contributions were defined by the sessions from the HOT Tasking Manager and constitute a subset of the overall contributions in OSM.

The spatial footprint of OSM contributions for building creations and highway creations was visually assessed using a map representation of the results. The maps were based on the equal-area Mollweide projection to allow for non-biased visual comparison between all geographic regions. The same symbology was applied to all maps to facilitate the detection of relative and absolute differences between overall and humanitarian mapping and between buildings and highways.

#### Cumulative distribution density analysis

Data from OSM and population has been aggregated to the subnational level to account for the spatial resolution of SHDI. We assess the cumulative distribution density of population and mapping in OSM in regard to four SHDI classes based on cutoff points defined by the United Nations Development Programme (UNDP)^44^: low human development (SHDI< 0.550), medium human development (SHDI: 0.550 - 0.699), high human development (SHDI: 0.700–0.799), very high human development (SHDI> 0.800).

#### GLM and spatial Eigenvector mapping

The relationship between SHDI, gender equality, disaster deaths, cellphone usage, number of YouthMappers chapters and population in regard to overall and humanitarian mapping contributions was analyzed at the sub-national scale based on a quasi-Poisson Generalized Linear Regression Model (GLM) with Spatial Eigenvector Mapping^24,45,46^. Using a count regression approach was necessary to account for the characteristics of the response variables which are count data. To account for overdispersion in the mean-variance relationship a quasi-poisson model was used.

It has been shown that there are important advantages of this approach over any other spatial modeling approach applied to geographical problems in the presence of spatial autocorrelation^24,45,46^. For instance, it has been shown that trend surface analysis might provide reasonable solutions, but only when the sample area is approximately homogeneous and the spatial structure to be modeled is rather simple and global, e.g. a gradient^24^. The required use of a quasi-poisson approach ruled out approaches such as generalized least squares, spatial lag or spatial error models that are only suitable for linear models^35^. Regression analysis without spatial eigenvector mapping led to strong spatial autocorrelation in the residuals. The spatial eigenvector mapping successfully removed spatial autocorrelation in the residuals. The regression analysis was performed in R^47^ using the packages spatialreg^48^, spdep^49^ and sf^50^. We performed this analysis for building creations and highway creations in OSM. The spatial weight matrix for the Spatial Eigenvector Mapping was based on a k-nearest-neighbor approach using the 10 closest neighbors and using row-standardized weighting. We evaluate the model’s performance by reporting estimates, standard error, z value and p value for each coefficient. Furthermore, the explained deviance is calculated to provide an estimate of the model’s power to explain the observed variability in the data set.

We assessed contribution inequality in respect to building creations and highways creations on the national level for all countries with low and medium human development (SHDI<0.7). For doing so the sub-national data set utilized previously was aggregated on the national level by summing up OSM contributions and population numbers. SHDI on the national level was derived as the weighted average of the sub-national index values considering population as the weight.

#### Temporal evolution

The analysis of the temporal evolution of humanitarian mapping activities was performed on a monthly basis based on a time series decomposition approach. The analysis relies on the mstl method implemented in R’s forecast package^51^. The time series is thereby decomposed into seasonal, trend and remainder components iteratively by loss smoothing^52^. Multiple seasonal periods were thereby allowed. In this article we provide a visualization of the observed values and the derived trend for humanitarian OSM contributions and the number of distinct mappers and validators in the HOT Tasking Manager.

We assessed the temporal dimension of contributions by deriving the similarity between time series for the cumulative distribution density of humanitarian building contribution per country using dynamic time warping (DTW)^53^. We relied on the implementation of the algorithm in python’s fastdtw library (https://github.com/slaypni/fastdtw). The similarity values were derived for all countries with low and medium human development (SHDI< 0.7). The analysis was complemented by an agglomerative hierarchical cluster analysis^26^ which defined five groups of countries. The distance matrix was based on the euclidean distance between DTW based distances for humanitarian OSM contributions for buildings. The analysis was conducted based on scikit-learn’s python implementation^54^ using the ward linkage criterion. Results were displayed for each cluster plotting the cumulative distribution density per country and highlighting the two countries with most overall contributions per group. As an addition to this the cluster dendogram was plotted.
